# Africa (COVID-19) Vaccine Technology Transfer: Where Are We?

**DOI:** 10.3390/life13091886

**Published:** 2023-09-09

**Authors:** Elijah Kolawole Oladipo, Seun Elijah Olufemi, Taiwo Ooreoluwa Ojo, Daniel Adewole Adediran, Akindele Felix Idowu, Usman Abiodun Idowu, Helen Onyeaka

**Affiliations:** 1 Department of Microbiology, Laboratory of Molecular Biology, Bioinformatics and Immunology, Adeleke University, P.M.B. 250, Ede 232104, Osun State, Nigeria; 2 Genomics Unit, Helix Biogen Institute, Ogbomoso 212102, Oyo State, Nigeria; oluwaseunjr1@gmail.com (S.E.O.); ojotaiwo718@gmail.com (T.O.O.); adedirandanieladewoleo4@gmail.com (D.A.A.); akindelefelix1@gmail.com (A.F.I.); idowuusmanabiodun@gmail.com (U.A.I.); 3 Department of Biochemistry, Ladoke Akintola University of Technology, Ogbomoso 210214, Oyo State, Nigeria; 4 Department of Pure and Applied Biology, Ladoke Akintola University of Technology, Ogbomoso 210214, Oyo State, Nigeria; 5 School of Chemical Engineering, University of Birmingham, Edgbaston, Birmingham B12 2TT, UK

**Keywords:** transfer, COVID-19, technology, vaccine, Africa

## Abstract

The rampant spread of the COVID-19 infection poses a grave and formidable challenge to global healthcare, with particular concern to the inhabitants of the African continent. In response to these pressing concerns, different strategies have been employed to combat the emergence of this insidious disease, encompassing crucial measures such as physical distancing, the utilization of face masks, meticulous hand hygiene, and widespread vaccination campaigns. Nevertheless, the economic realities faced by numerous African nations, characterized by their classification as “low-income countries (LICs)”, present a formidable barrier to accessing and distributing approved vaccines to their populations. Moreover, it is essential to discuss the hesitancy of the European Union (EU) in releasing intellectual property rights associated with the transfer of vaccine technology to Africa. While the EU has been a key player in global efforts to combat the pandemic, there has been reluctance in sharing valuable knowledge and resources with African countries. This hesitancy raises concerns about equitable vaccine access and the potential for a prolonged health crisis in Africa. This review underscores the urgent imperative and need of establishing localized vaccine development and production facilities within Africa, necessitating the active involvement of governments and collaborative partnerships to achieve this crucial objective. Furthermore, this review advocates for the exploration of viable avenues for the transfer of vaccine technology as a means to facilitate equitable vaccine access across the African continent and also the cruciality and the need for the EU to reconsider its stance and actively engage in transferring vaccine technology to Africa through sharing intellectual property. The EU can contribute to the establishment of localized vaccine production facilities on the continent, which will not only increase vaccine availability but also promote self-sufficiency and resilience in the face of future health emergencies.

## 1. Introduction

The COVID-19 pandemic, an all-encompassing global threat, has left no region untouched in the entire world [1]. As of 9 July 2023, the tally of confirmed COVID-19 cases stands at a staggering of 767,972,961, with a heart-wrenching death toll of approximately 6,950,655 worldwide [2]. Data profiles from the World Health Organization (WHO) in data records, as shown in Table 1, further shows the recorded cases across the population, as alarming reports continue to pour in regarding the surge of COVID-19 cases in low- and middle-income countries (LMICs) like Bangladesh, Cameroon, the Democratic Republic of Congo, India, Indonesia, Nigeria, Mali, Gambia, Senegal, Ghana, Sudan, Tunisia, and various other African nations [3,4]. Africa, in particular, has confirmed cases of 9,542,363 and the death toll reaching 175,399 as of 9 July 2023. Tragically, the scarcity or absence of COVID-19 vaccines in many nations has translated into an escalating number of lives lost. In the initial stages of the pandemic, when neither a COVID-19 vaccine nor treatment options were known, the concept of herd immunity emerged as a potential remedy for combating the SARS-CoV-2 virus [1].

Amidst this relentless surge, multiple nations have made significant strides in developing vaccines against the disease. Notably, certain high-income countries (HICs) have made remarkable progress in the manufacturing and development of COVID-19 vaccines [5]. Concurrently, numerous vaccine candidates have received emergency use authorizations, leading some countries to commence extensive vaccination campaigns [5]. However, the mere existence of licensed vaccines falls short of achieving global control over COVID-19. It is imperative to ramp up production on a larger scale, ensuring affordability, broad coverage, and equitable distribution across the global population. This encompasses addressing the evolving genetic makeup of the virus, particularly in Africa, where unique host—virus interactions have given rise to new variants [6]. Consequently, the World Health Organization (WHO) and its collaborative partners have diligently worked towards enhancing worldwide access to COVID-19 vaccines. Their efforts involve bolstering the capabilities of low- and middle-income countries (LMICs) to facilitate vaccine production, thereby curbing the pandemic’s impact [7].

To facilitate the manufacturing process of LMICs, the WHO is launching a project in South Africa, providing technical expertise for vaccine production, empowering the continent to combat the epidemic, and attain self-sufficiency [8]. The success of vaccine technology transfer to Africa hinges upon the region’s preparedness to embrace this knowledge hub, with far-reaching implications for the vaccine’s efficacy. The implementation of effective strategies is essential to overcome barriers hindering the seamless transmission of expertise.

This review emphasises the European Union’s commitment to protecting intellectual property rights during the transfer of vaccine technology. It also highlights the ethical and equity challenges surrounding the technology transfer in Africa and also provides insights into the efforts needed from African governments and unions to facilitate the transfer of vaccine technology, enabling the region to effectively combat COVID-19 and emerging infectious diseases, including their variants. Implementing the recommended measures can contribute to strengthening Africa’s response to public health crises while ensuring equitable access to life-saving vaccines.

## 2. Rationale for COVID-19 Vaccine Technology Transfer in Africa

On 11 March 2020, the World Health Organization (WHO) officially declared the COVID-19 pandemic, marking a turning point in global health [9]. Notably, as of 18 November 2022, Africa accounted for 3.48% of the total worldwide infections, with 12,693,549 confirmed cases. South Africa bore the brunt of the outbreak on the African continent, reporting approximately 4,036,623 infections, making it the most heavily impacted country on the continent [10]. It is important to note that Africa’s lower number of confirmed cases and death rates compared to other continents may be attributed to limited COVID-19 testing, which skews the incidence rate and fails to provide an accurate depiction of the outbreak’s magnitude [9].

Infectious diseases remain a leading cause of death in Africa and Asia, particularly among children under the age of five. In 2016, Sub-Saharan African countries accounted for 44.4% of all child fatalities, while South Asian countries accounted for 248%. Shockingly, children in low- and middle-income nations face a mortality risk 34 times higher than their counterparts in high-income countries [11]. This underscores the urgent need for targeted interventions and healthcare improvements to safeguard the well-being of vulnerable populations in these regions.

**Figure 1:**
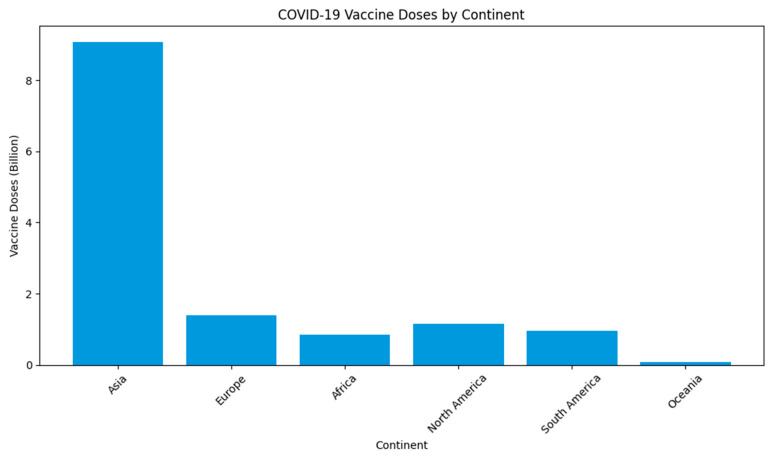
Total COVID-19 vaccine doses (billion) across the continent. (Source: our world in data, accessed on 25 July 2023, https://ourworldindata.org/).

At the end of June 2023, an estimation of 13 billion vaccine doses had been administered worldwide, with a meagre 0.2% allocated to low-income countries, especially in Africa and the Oceania continent, as depicted in Figure 1 below [12]. WHO statistics, as reported by Id and colleagues [3] in 2020, revealed the ongoing spread of COVID-19 in densely populated regions of Ghana, Nigeria, the Democratic Republic of Congo, and potentially across the African continent. This emphasises the imperative of a global commitment to developing a safe and affordable COVID-19 vaccine, with a particular emphasis on prioritizing vaccination efforts in African nations. Dr. Seth Berkley, the CEO of GAVI (the Vaccine Alliance), has underscored the crucial importance of addressing the specific vaccine needs of these African countries, making it an urgent global priority.

In 2021, the World Health Organization (WHO) established a consortium in South Africa to establish the inaugural COVID mRNA vaccine technology transfer hub [13]. Vaccine production entails various challenges, such as process development, maintenance, lead time, manufacturing facilities, equipment, life cycle management, and product portfolio management [11]. The WHO highlights the critical importance of a robust and stable production process, as well as consistent component supply over an extended period, ensuring the vaccine’s longevity in the market. When considering investments in vaccine production in Africa, a careful evaluation of these factors is essential. Neglecting to address these risks may result in costly product recalls, market suspensions, and penalties if a manufacturer fails to fulfil supply agreements.

In a study by Asundi in 2020 [14], the advancement of next-generation vaccines based on mRNA, viral vector, or protein subunit technologies presents promising opportunities to alleviate the impact of infectious diseases. The utilisation of these technologies during the COVID-19 pandemic demonstrated the speed at which new vaccines can be developed. However, challenges arise when it comes to scaling up vaccine production to achieve global vaccine equity, highlighting the obstacles that these innovative technologies still face in meeting global demand. In medical technology development, legal barriers such as intellectual property transfer are widely recognized.

In collaborative efforts with Biovac, Afrigen Biologics and Vaccines, a network of universities, and the Africa Centers for Disease Control and Prevention (ACDC), the WHO and its COVAX partners are actively working towards establishing the first COVID-19 mRNA vaccine technology transfer hub in South Africa [13].

The establishment of vaccine production capacity, including for COVID-19, presents unique challenges in Africa. Many African countries encounter obstacles such as limited knowledge, scarcity of raw materials, consumables, and equipment, market access restrictions, import policies, regulatory limitations in good manufacturing practice (GMP) inspection, and lengthy delays in dossier review and clearance [11]. Additionally, essential factors such as facility development, financial support, and technology acquisition must be considered. These challenges are compounded by other prevalent issues in Africa, such as inadequate infrastructure, a shortage of healthcare professionals, diverse religious landscapes, and uneven population distribution [15].

## 3. The Ongoing Endeavours in Africa to Develop Domestically Manufactured and Approved COVID-19 Vaccines

More than 80% of Africa’s population remains unvaccinated, largely due to the unequal availability of vaccine production, of which the available ones are concentrated in a few high-income nations in Africa [16]. The COVID-19 pandemic shed light on Africa’s heavy reliance on imported immunizations, with 99% of vaccines being sourced externally from other parts of the world [17]. Consequently, African governments have faced challenges in vaccinating their populations. The news of the German biotechnology company BioNTech establishing COVID-19 vaccine manufacturing facilities in Rwanda and Senegal brought hope to many African countries [18].

Inadequate and unreliable vaccine supply, coupled with unequal distribution, poor delivery networks, and infrastructure limitations, have hindered mass vaccination efforts across the continent. Transferring vaccine technology to African countries is seen as a positive step toward enhancing immunisation coverage throughout Africa [18]. Some African countries are actively working on developing their own approved vaccines, aiming to improve self-sufficiency [19]. Currently, only 10 African vaccine manufacturers are situated in five countries: Egypt, Morocco, Senegal, South Africa, and Tunisia. However, the majority of local businesses in Africa focus on packaging, labelling, and occasionally filling and finishing, with limited upstream manufacturing capabilities [20].

To address these challenges, the US International Development Finance Corporation, in collaboration with European partners, announced a EUR 600 million (USD 710 million) financing package for South Africa’s Aspen Pharmacare. By the end of 2023, Aspen’s factory is expected to produce millions of doses and engage in the “fill-and-finish” process for approximately 500 million Johnson & Johnson doses [21]. Additionally, South Africa’s Biovac Institute has taken the responsibility of accelerating the Pfizer vaccine fill-and-finish production in Cape Town, starting in 2022. In Senegal, the government is partnering with the Foundation Institute Pasteur de Dakar, with support from the US and Europe, to establish a USD 200 million COVID-19 vaccine manufacturing facility that will encompass both the production of vaccine substances and fill-and-finish operations [21]. Moreover, Egypt has plans to manufacture Chinese Sinovac at a new Vacsera facility near Cairo, with a capacity of 1 billion vaccines per year. Egypt is also exploring agreements for drug substance manufacture and fill-and-finish of Russia’s Sputnik V vaccine, aiming to reduce Africa’s reliance on vaccine imports [21].

The Coalition for Epidemic Preparedness Innovations (CEPI) and the Institute Pasteur de Dakar (IPD) have formed a partnership to promote equitable access to vaccines in Africa and support the African Union’s goal of increasing the share of vaccine supply from African manufacturers to 60% by 2040. This collaboration aims not only to establish COVID-19 vaccines but also advance vaccine development for other diseases [22].

## 4. Challenges and Obstacles Faced by Africa in Establishing Vaccine Producing Industry

Vaccines, known for their cost-effectiveness in controlling and preventing infectious diseases, have seen limited development efforts in Africa in recent decades [23]. The continent heavily relies on imported medications, with approximately 70–80% of medicines being imported [19]. Unfortunately, vaccine production in Africa has been insufficient, accounting for less than 1% of the vaccines used across the continent. Some local producers have ceased production due to their inability to compete with imported vaccines, resulting in obsolescence. Africa has only a handful of vaccine makers [19]. Furthermore, as vaccines manufactured in India are redirected for domestic use, African countries relying on doses from COVAX and the Serum Institute of India face delays, contributing to Africa receiving only 1% of the global COVID-19 vaccine distribution, despite having 17% of the world’s population [24].

African countries, particularly low-income ones, face challenges related to reciprocity following vaccine trials in their populations and the need for local vaccine manufacturing. Access to SARS laboratory facilities is also a concern for scaling up testing and sequencing for local SARS and detecting variants of the SARS-CoV-2 virus in Africa [25]. To address these issues, countries must increase funding for vaccine research, improve access, strengthen storage infrastructure, maintain an efficient cold chain, ensure effective pharmacovigilance, and tackle vaccine hesitancy through community education regarding the vaccines’ benefits, safety, side effects, lack of trust in pharmaceutical industries, and media misinformation [26]. It is also crucial to identify priority populations for vaccination [25].

According to Foresight Africa 2022, the challenge in Africa is no longer solely about vaccine supply but rather the unequal distribution of vaccines across the continent. Despite global vaccine production steadily increasing at a rate of approximately 1.5 billion doses per month, Africa has received only a small portion of the global supply. By January, Africa had received about 540 million doses (approximately 6% of all COVID-19 vaccinations) and administered 309 million doses out of over 9 billion vaccine doses manufactured worldwide, correlating to only about 10% of the African population being properly immunised [27].

Insufficient resources pose one of the most significant issues for the African continent. Prior to launching any immunisation program, adequate funding is required for widespread distribution. For instance, the cost of fully immunising a single child in Africa with the DTP vaccine (diphtheria, tetanus, and pertussis) ranges from USD 25 to USD 45, excluding additional logistics costs. Despite the overall positive impact, African leaders often allocate limited finances to immunisation efforts in their respective countries [28]. Currently, over USD 12 billion is needed for the COVID-19 immunisation exercise in Africa, including the purchase of over 1.4 billion doses to vaccinate 60% of the population and achieve herd immunity [28,29]. However, the financial capacity of most African countries has been severely impacted by the pandemic crisis, making it challenging to secure the necessary funding for immunisation [28].

The requirement for a substantial upfront investment and limited financial support within the continent make repurposing facilities for large-scale vaccine production complex. According to a study conducted by the African Vaccine Manufacturing Initiative (AVMI), the World Health Organization (WHO), and the United Nations Industrial Development Organization (UNIDO), the cost of constructing a vaccine manufacturing plant in Africa ranges from USD 60 million to USD 130 million [19].

Africa’s limited participation poses a significant obstacle in developing vaccine industries. The Africa Centre for Disease Control and Prevention (Africa CDC) has played a crucial role in managing the COVID-19 response, emphasising the need to increase Africa’s involvement in research and development. Currently, Africa accounts for only 2% of all global clinical trials, and increased investment in research and development is necessary to enhance vaccination efficacy across the continent [29].

A review of COVID-19 broadcast messaging in African countries revealed misinformation and concealing of the truth about the virus, leading to public distrust, antagonism, and suspicion towards health officials. Ambiguous signals often result in individuals being misinformed and overlooking important information. Interestingly, the majority of public awareness messaging discourages animal contact and COVID-19 vaccine uptake [30].

Storage is another critical issue that needs to be addressed. Vaccination programs in Africa have long struggled with inadequate storage systems due to limited funds for power supply, cold chain maintenance, and organised vaccine shipment monitoring. This has led to product expiration and inactivation in many impoverished countries. Challenges such as erratic electricity, frequent power outages, limited cold chain equipment and facilities, poor road networks hindering vaccine distribution, untrained vaccinators, and a lack of basic amenities like clean water and housing contribute to these storage difficulties, especially in rural communities and villages [28].

## 5. Challenges of Intellectual Property Faced in the Transfer of COVID-19 Vaccine Technology to Africa

The transfer of COVID-19 vaccine technology doesn’t face challenges only due to issues related to problems from the African government but also due to the transfer of intellectual property (IP) rights [31]. The proposal for a temporary waiver of IP protections faced stringent opposition from owner nations, especially the European Union, which has hindered progress towards achieving equitable access to vaccines on the continent. However, there have been recent developments, and efforts have brought hope for positive change and potential solutions to this menace. One of the major obstacles in advancing the proposal for an IP waiver is the divergence of views on its scope by different countries. Proponents of the waiver seek to suspend intellectual property protections for all COVID-19 health technologies including vaccines, medicines, diagnostics, and personal protective equipment [32]. However, some countries express concerns about the broad scope of the waiver as they argue that alternative measures, such as increasing vaccine supplies through other means, should be explored instead. Finding common ground and addressing these concerns is vital to making progress in the transfer of this technology to Africa [33].

Technology transfer and the sharing of know-how are critical components for effective vaccine production. In addition to intellectual property waivers, developing countries need access to essential materials and knowledge from original manufacturers and research institutes. Collaborative mechanisms like the WHO COVID-19 Technology Access Pool (C-TAP) have been playing a pivotal role in facilitating technology transfer and knowledge sharing among countries [21]. In the effort of leveraging these collaborative platforms, African nations can acquire the necessary expertise and resources to enhance their technical know-how of vaccine manufacturing capabilities, but this collaborative effort has been plummeting due to the hesitancy of the nations with the intellectual property. Ethical considerations and the principle of equity are central to the discussions surrounding intellectual property challenges. Striking a balance between protecting intellectual property rights and meeting global health needs is paramount [34]. While an IP waiver could potentially drive down vaccine costs and increase access, opponents argue that it may disrupt the global supply chain and divert scarce resources. Finding a solution that addresses these concerns while ensuring equitable access to vaccines is crucial to move beyond when faced with an emergent or new infectious disease [35].

African leaders have been at the forefront of advocating for technology transfer and equitable access to vaccines. The African Union’s Vaccine Acquisition Task Team (AVATT) has spearheaded efforts to secure vaccine supplies and build local manufacturing capacity [36]. The recent support from the Biden administration for the IP waiver, as well as the mounting pressure on opposing countries, has injected renewed hope for progress in the transfer of the technology. It is imperative that African leaders maintain their proactive stance, working in collaboration with global health organizations and international partners to ensure that technology transfer initiatives align with Africa’s priorities and contribute to overcoming the vaccine shortage on the continent [37].

## 6. Ethical Considerations and Equity in Vaccine Technology Transfer

The transfer of COVID-19 vaccine technology to low-income countries also presents ethical challenges and raises questions about equity in global health. It is crucial to prioritize fair access to life-saving vaccines for all populations, regardless of their socioeconomic status or geographic location [38]. We explore the ethical considerations and equity issues surrounding vaccine technology transfer. Ethical considerations in vaccine technology transfer encompass multiple dimensions. Distributive justice, a principle rooted in ethics, demands fair allocation of limited resources, including vaccines. Achieving equity requires ensuring that no population is left behind in the pursuit of vaccination [39]. Additionally, ethical practices dictate that informed consent is obtained, participant rights are protected, and adherence to ethical research standards is maintained. Transparency in collaboration, knowledge sharing, and technology transfer processes is essential to foster trust and uphold autonomy [40].

Equity in vaccine technology transfer requires addressing disparities in access, resources, and capabilities. Low-income countries often face challenges related to infrastructure and resources necessary for effective vaccine production. To ensure equity, technology transfer initiatives should include capacity-building measures such as knowledge sharing, technical assistance, and support for local manufacturing [41]. Collaboration between governments, pharmaceutical companies, and international organizations is crucial to overcome barriers and promote sustainable vaccine production in resource-limited settings. However, achieving equity in vaccine technology transfer comes with its own set of challenges. Intellectual property barriers, limited access to research and development expertise, and economic disparities pose significant obstacles [14]. To overcome these challenges, stakeholders must consider implementing intellectual property waivers to facilitate technology transfer. Additionally, expanding technology transfer programs and providing financial and technical support to low-income countries can help bridge the gaps. Collaborative platforms such as the WHO COVID-19 Technology Access Pool (C-TAP) play a crucial role in facilitating the equitable sharing of knowledge, resources, and expertise [42].

Ethical considerations and equity are vital components of vaccine technology transfer. By prioritizing fair access, transparency, and collaboration, we can promote the equitable distribution of life-saving vaccines [39]. Efforts should focus on removing barriers, fostering technology transfer, and empowering low-income countries to strengthen their vaccine production capacities. Ultimately, these endeavors save lives and contribute to achieving global health equity.

## 7. Recommendations

In the journey towards protecting their people from vaccine-preventable diseases, African health systems have made remarkable strides. They have diligently implemented routine immunization plans, improved emergency disease response, and achieved significant milestones like the eradication of polio. This dedication and progress reflect the continent’s unwavering commitment to safeguarding the well-being of its population, a commitment that is further exemplified in its response to the COVID-19 pandemic. However, despite the World Health Organization’s statement on 21 June 2021, progress toward equitable vaccine access has been disappointingly slow. It is crucial for governments and organizations like GAVI and the Vaccine Alliance to join forces, fostering a spirit of cooperation, collaboration, and fairness. Together, they must strive to establish a system that ensures timely and unrestricted access to COVID-19 vaccines, leaving no room for monopolies.

The emergence of new virus strains and the escalating number of daily cases present African countries with a daunting challenge. Waiting for months, or even years, to gain access to vaccines may prove to be a daunting and impractical task. Therefore, it becomes imperative for African governments to go beyond vaccine transfer rather the technology transfer. They must intensify their efforts in combating COVID-19 by allocating adequate financial resources, fostering the creation of accurate data and projections, and enhancing national immunization program coordination. These collective actions will empower African nations to actively participate in the global partnership for COVID-19 vaccines, ensuring that their people are not left behind.

In this crucial fight against the pandemic, it is the responsibility of each African government to rise up and support their local scientists who are passionate about bringing favorable solutions in the fight against pandemics. They should forge partnerships with nations owning intellectual property in order to achieve equitable access to expand vaccine development capacity and the capacity building of earlier career researchers across the African continent. By embracing this united front, African countries may not only address the immediate challenges posed by COVID-19 but also lay the foundation for a brighter and healthier future for their citizens in case of future exposure to other diseases that can be combated with vaccines.

## 8. Conclusion

Despite the reduced level of COVID-19 cases and incidence in Africa and the world at large recently, there is a need for preparing for the possible reemergence of its different variant strains or some other virus infections in the future. The establishment of vaccine manufacturing industries in Africa is a necessity to lower vaccine costs, assist in overcoming vaccine shortages, improve the countries’ ability to respond to outbreaks, and provide a path for African countries to gain independence from large pharmaceutical companies. Furthermore, African governments should take charge of this responsibility and form collaborations with initiatives in developing indigenous innovations to combat COVID-19 in Africa in order to maintain pan-African goals.

## Figures and Tables

**Table 1:** COVID-19 cases in each continent and the respective population size as of 16 August 2023.

WHO Regions	Total Cases (Million)	Population (Billion)
Europe	249.09	0.74481
Asia	299.92	4.72
North America	124.41	0.60032
South America	68.8	0.43882
Africa	13.11	1.43
Oceania	14.43	0.04504

## Data Availability

Not applicable.
